# The impact of SARS-CoV-2 pandemic on suicidal ideation admissions: a single adolescent and young adult psychiatry center experience

**DOI:** 10.1192/j.eurpsy.2022.828

**Published:** 2022-09-01

**Authors:** D. Vila-Chã, B. Leal, I. Pinto, R. Mateiro, M. Avelino, J. Salgado

**Affiliations:** Centro Hospitalar Psiquiátrico de Lisboa, Psiquiatria, Lisboa, Portugal

**Keywords:** suicidal ideation, pandemic, SARS-CoV-2

## Abstract

**Introduction:**

The COVID-19 pandemic and the lockdown brought about a sense of fear and anxiety around the globe. This phenomenon led to both short and long term psychosocial and mental health implications for children and adolescents.

**Objectives:**

To evaluate the effect of COVID-19 pandemic on hospital admissions for suicidal ideation in a Portuguese adolescent and young adult psychiatry service.

**Methods:**

We conducted a single-center, retrospective study including adolescent and young adult patients (15-25 years old) admitted to our service with suicidal ideation within a year before and a year after the declaration date of SARS-CoV-2 disease as a pandemic. Patients were divided in two groups: Group A - patients admitted before the pandemic (March 11, 2019 and March 11, 2020) and Group B – patients admitted after the pandemic declaration (March 12, 2020 and March 12, 2021). The groups characteristics and outcomes were assessed and compared.

**Results:**

A total of 647 admissions were assessed (Group A, n= 372 and Group B, n=275). Demographic characteristics were similar between groups. 75 patients (vs 25 patients) were admitted with suicidal ideation in the year before the pandemic. There was a lower proportion of patients admitted with suicidal ideation during the year after the pandemic year - OR 0.374 (95% CI 0.228-0.614, P<0.001).

**Conclusions:**

Our study showed a decrease in admissions for suicidal ideation in this service in the year after the pandemic. More studies are needed to understand the factors that may justify this decline and evaluate the longer effects of this pandemic in mental health.

**Disclosure:**

No significant relationships.

